# Clinical evaluation and treatment of obesity in women across selected life stages: a narrative review

**DOI:** 10.4069/whn.2026.09.02

**Published:** 2026-09-29

**Authors:** Yongho Lee, Bumjo Oh

**Affiliations:** 1 Department of Family Medicine, SMG-SNU Boramae Medical Center, Seoul, Korea; 2 School of Transdisciplinary Innovations, Seoul National University, Seoul, Korea

**Keywords:** Glucagon-like peptide-1 receptor agonists, Menopause, Obesity, Polycystic ovary syndrome, Women’s health

## Abstract

**Purpose:**

This study was performed to synthesize current evidence on evaluating and treating obesity in women across selected life stages relevant to women’s health nursing practice.

**Methods:**

For this invited narrative review, targeted searches of PubMed and guideline and regulatory sources available through July 2026 identified current diagnostic and staging frameworks, Korean and international guidance, pharmacotherapy evidence, official prescribing information, and literature on reproductive and menopausal physiology for synthesis. Sources were selected purposively rather than systematically.

**Results:**

The Lancet criteria for clinical obesity, the Edmonton Obesity Staging System, and a four-domain assessment of mechanical, metabolic, mental, and social milieu factors offer complementary ways to confirm adiposity, diagnose illness, stage severity, and assess health beyond body mass index. Medication selection should be based primarily on approved indications, efficacy, safety, comorbidities, reproductive plans, access, and patient preferences; phenotype-guided matching remains investigational. Care for women of reproductive age should address polycystic ovary syndrome, pregnancy intention, and the oral hormonal contraceptive precautions specific to tirzepatide. During pregnancy and lactation, appropriate weight trajectories and medication safety for the individual agent take precedence over pharmacologic weight loss. Declining estradiol during the menopausal transition is associated with central fat redistribution, while lean mass loss also reflects aging; an independent effect of follicle-stimulating hormone remains unestablished. Menopausal hormone therapy should be prescribed for established menopausal indications rather than for weight loss.

**Conclusion:**

Obesity care for women should integrate disease-based assessment, individualized treatment supported by evidence, medication counseling tailored to life stage, nonstigmatizing communication, and shared decision-making. Nurses should support assessment, education, monitoring, and coordination within their scope of practice.

## Introduction

An imbalance of calories or a concern about appearance no longer adequately describes obesity. Recognition of obesity as a chronic, relapsing disease with organ-level consequences is increasing; if unaddressed, it compounds the risk of type 2 diabetes, cardiovascular disease, certain cancers, and premature death [1]. In Korea, 38.4% of adults had obesity in 2022, and the prevalence of class 2 and class 3 obesity had increased 1.6-fold and 2.6-fold, respectively, since 2013 [2].

A single, static framework cannot adequately describe obesity in women. Reproductive endocrine changes are accompanied by variation in body composition, fat distribution, and metabolic risk across reproductive and menopausal stages [3,4]. Pregnancy and lactation further change weight trajectories and treatment priorities, as discussed below [5-8]. A woman’s risk profile, the safety of pharmacologic treatment, and even the pathophysiology underlying her weight gain vary substantially with her current life stage. Nurses and other health professionals who care for women during health check-ups, in primary care, in obstetric and gynecologic settings, and in chronic disease clinics are therefore uniquely positioned to recognize and address these patterns of risk. Yet much of the available clinical guidance addresses a generic adult patient rather than the specific physiology of women across life stages.

Developed from a continuing education lecture delivered at the 2026 Spring Conference of the Korean Society of Women Health Nursing, this invited review has four aims: (1) summarize the shift from a weight-centric to a disease-centric model for defining and diagnosing obesity; (2) describe a structured framework for comprehensive clinical assessment; (3) critically appraise phenotype-guided pharmacotherapy as an emerging precision approach that is not yet established; and (4) examine how obesity assessment and treatment should be adapted to three selected female life stages most relevant to women’s health nursing practice—the reproductive years, pregnancy and lactation, and the menopausal transition. Figure 1 summarizes considerations specific to these stages and their nursing implications. A clinical case illustrates the application of this framework throughout the review. The review is therefore limited to selected reproductive-to-menopausal contexts; adolescent obesity and geriatric obesity in later life require separate, age-specific consideration and are not comprehensively covered here.

Targeted searches of PubMed, major clinical practice guidelines, and official prescribing information available through July 2026 were used to identify relevant literature. Search terms combined obesity assessment, diagnosis, staging, and pharmacotherapy with reproductive-age women, polycystic ovary syndrome (PCOS), pregnancy, lactation, and menopause. Recent guidelines, regulatory documents, landmark randomized trials, and studies directly addressing obesity assessment and treatment across these life stages were prioritized; Korean guidance was included when national practice differed from international recommendations. For this invited narrative review, studies were selected purposively rather than systematically or exhaustively, and neither formal risk-of-bias appraisal nor quantitative synthesis was undertaken.

## Redefining obesity: from body weight to chronic disease

For decades, body mass index (BMI) alone has served as the operational definition of obesity. Although inexpensive and reproducible, BMI cannot distinguish fat from muscle, characterize adipose tissue distribution, and its relationship to adiposity varies across age, sex, and ethnic groups [1]. A bodybuilder with a high BMI but little visceral fat may be misclassified as having obesity, whereas a woman with excess central adiposity and a BMI below the obesity threshold—normal-BMI excess adiposity—may be overlooked entirely. In Korean clinical practice, BMI and waist circumference remain practical measures for classifying obesity and abdominal obesity. Obesity is classified as class 1 (BMI of 25.0–29.9 kg/m^2^), class 2 (30.0–34.9 kg/m^2^), or class 3 (≥35.0 kg/m^2^), and abdominal obesity is defined by a waist circumference ≥90 cm in men or ≥85 cm in women [9]. Where available, body composition measurements provide information about fat mass and its distribution that BMI cannot capture [9].

BMI also fails to convey clinical severity. BMI does not consistently reflect disease burden: a patient with a BMI of 37 kg/m^2^ may have no complications and preserved function, whereas one with a BMI of only 28 kg/m^2^ may already have central adiposity and severe metabolic disease. In recognition of this limitation, the World Health Organization described obesity in 2000 as a complex, multifactorial condition requiring long-term prevention and management [10]. Current understanding centers on dysregulation of appetite, satiety, reward, and energy-expenditure pathways and their interaction with genetic, environmental, and psychosocial factors, rather than a simple failure of willpower [1,10]. Weight-related stigma during clinical encounters can deter patients from seeking care and compromise its quality, underscoring the need for respectful communication informed by an understanding of the disease [11,12].

In 2025, the *Lancet Diabetes & Endocrinology* Commission on Clinical Obesity proposed a two-part diagnostic process. The first step is to confirm excess adiposity through direct body fat measurement, when available, or BMI plus at least one additional anthropometric measure, such as waist circumference, waist-to-hip ratio, or waist-to-height ratio; excess adiposity may be pragmatically assumed when BMI exceeds 40 kg/m^2^ [13]. The second step is to diagnose clinical obesity, which requires confirmed excess adiposity together with obesity-attributable signs or symptoms of ongoing organ or tissue dysfunction, substantial age-adjusted limitations in mobility or basic activities of daily living, or both [13]. Preclinical obesity denotes confirmed excess adiposity with preserved organ function and no such activity limitation, although future health risk may still be increased. Confirming adiposity alone therefore does not establish a disease diagnosis, nor does an unrelated comorbidity establish clinical obesity: the clinical manifestation must be attributable to excess adiposity [13].

### Case, part 1

This composite case was constructed for educational illustration and does not describe a single identifiable patient.

A 58-year-old woman who was 162 cm tall and weighed 72 kg (BMI, 27.4 kg/m^2^) presented six years after menopause. She was receiving medication for dyslipidemia. Bioelectrical impedance analysis (BIA) estimated her body fat percentage at 38%, and her waist circumference of 88 cm exceeded the abdominal obesity threshold. Over the preceding three years, she had gained 11 kg, with a body composition pattern suggesting loss of lean mass and an increase in estimated visceral adiposity. Her fasting glucose level was 108 mg/dL, and her triglyceride level was 158 mg/dL. She reported exercising regularly but could not understand why she continued to gain weight. Under Korean criteria, BMI alone would place her in class 1 obesity; her waist circumference, body composition, and emerging metabolic abnormalities, however, indicated a substantially more concerning clinical picture that BMI alone would have missed. She satisfies the first step of the two-part diagnostic process described above: a BMI above the Korean obesity threshold, a waist circumference of 88 cm, and a body fat percentage of 38% confirm excess adiposity. For the second step, her impaired fasting glucose (108 mg/dL) and hypertriglyceridemia (158 mg/dL) satisfy the Commission’s metabolic criterion of hyperglycemia or glucose intolerance with an abnormal lipid profile, provided that clinical judgment attributes these abnormalities to excess adiposity; her mobility and basic activities of daily living remain preserved. On this basis, she would be classified as having clinical rather than preclinical obesity [13]. Because attribution depends on clinical judgment rather than a test result, alternative contributors, such as familial dyslipidemia, should still be considered. Case, part 2 addresses severity after introducing the staging framework.

## Comprehensive clinical assessment: confirm adiposity, diagnose, then stage severity

The Lancet diagnostic criteria, the Edmonton Obesity Staging System (EOSS), and the four-domain assessment of mechanical, metabolic, mental, and social milieu factors (4Ms) address different clinical questions. The Lancet criteria establish whether confirmed excess adiposity has caused current illness. EOSS grades the overall obesity-related medical, mental, and functional burden across five levels (stages 0–4): no apparent risk factors or impairment (stage 0); subclinical risk factors or mild symptoms (stage 1); established obesity-related chronic disease or moderate limitation (stage 2); significant end-organ damage or functional impairment (stage 3); and severe, potentially end-stage disease (stage 4) [14]. As a severity-staging tool, EOSS does not replace the Lancet diagnostic criteria. The 4Ms organize assessment to help clinicians identify obesity-related consequences, root causes, and barriers to management [15]. This assessment mnemonic complements diagnosis and EOSS staging; it is neither a diagnostic nor a severity score.

These frameworks can be adapted into a practical sequence for Korean outpatient and health check-up settings: (1) confirm excess adiposity with BMI plus waist circumference or another anthropometric measure, or direct body fat measurement where available; (2) determine whether obesity-attributable organ or tissue dysfunction or age-adjusted limitations in mobility or basic activities of daily living meet the Lancet criteria for clinical obesity; (3) use the 4Ms to characterize mechanical and metabolic consequences, mental health, social context, root causes, and treatment barriers; (4) grade overall severity with EOSS; and (5) agree on treatment intensity, referral needs, and follow-up. Derived from the cited frameworks, this sequence is an implementation aid rather than a separately validated diagnostic instrument. Nurses can support the process by obtaining accurate anthropometric and body composition measurements and identifying symptoms or functional limitations that require further evaluation.

## Setting the treatment goal: clinically meaningful, not maximal, weight loss

After severity staging, a realistic weight-loss target should be set on the basis of evidence. Korean clinical practice guidelines recommend that most patients starting treatment initially aim to lose 5%–10% of their baseline weight within 6 months [16]. The benefits associated with this target should not be attributed to weight loss alone: in the Diabetes Prevention Program, an intensive lifestyle intervention targeting at least 7% weight loss and at least 150 minutes of physical activity per week reduced type 2 diabetes incidence by 58% relative to placebo over a mean follow-up of 2.8 years in adults with impaired glucose tolerance [17]. In the Look AHEAD trial of adults with type 2 diabetes, clinically meaningful improvements in blood pressure, lipid profile, and glycemic control were associated with weight loss of 5%–10% [18]. A 5%–10% weight reduction is clinically meaningful because it can improve cardiometabolic risk factors and some obesity-related complications, although the magnitude of benefit depends on the condition and baseline risk [16,18]. Clinicians and patients should therefore focus first on weight loss that measurably improves health rather than on reaching an “ideal body weight.”

Current Korean guidelines indicate pharmacotherapy for patients with a BMI ≥25 kg/m^2^ who have not lost adequate weight through lifestyle modification alone, using agents supported by long-term, large-scale clinical trials [16]. Eligibility under guidelines differs from an approved indication: Korean guidelines consider pharmacotherapy at a BMI ≥25 kg/m^2^, whereas currently available agents are approved for a BMI ≥30 kg/m^2^ or ≥27 kg/m^2^ with at least one weight-related comorbidity. Prescribing should satisfy both criteria [9]. Response should be formally assessed three months after the patient reaches a maintenance dose: those who have lost at least 5% of their weight are considered responders and continue the regimen, whereas those below this threshold are candidates for dose adjustment or a different agent. Antiobesity medications approved for long-term treatment may be continued to support weight maintenance [16].

## Individualized pharmacotherapy: established factors first, phenotyping exploratory

No single antiobesity medication is optimal for every patient. Selection should begin with the approved indications, expected efficacy and safety, obesity-related comorbidities, contraindications, concomitant medications, reproductive plans and life stage, access, cost, and patient preferences [9,16]. Although eating behavior and metabolic characteristics may inform counseling and generate hypotheses, current evidence does not establish a validated algorithm for matching treatment to phenotype as standard care.

A frequently cited study classified obesity into four physiological phenotypes: “hungry brain” (abnormal satiation), “emotional hunger” (hedonic eating), “hungry gut” (abnormal satiety), and “slow burn” (reduced resting energy expenditure). At 12 months, it reported greater weight loss with phenotype-guided treatment than with standard medical weight management (15.9% vs. 9.0%) [19]. A subsequent correction stated that treatment allocation had not been randomized and that the study was best characterized as an observational comparison of two convenience samples [20]. The comparison was conducted at a single center without blinding, involved a small sample with limited diversity, and was susceptible to baseline and testing bias. The findings therefore generate hypotheses that require testing in larger, multicenter randomized trials [19,20]. Meanwhile, glucagon-like peptide-1 (GLP-1) receptor agonists and dual GLP-1/glucose-dependent insulinotropic polypeptide (GIP) receptor agonists have become central to modern pharmacotherapy. Randomized trials reported mean weight reductions of approximately 15% with semaglutide [21] and over 20% with tirzepatide [22] but did not test phenotype-matched prescribing. The choice of agent, dose, and adjunctive lifestyle strategy should therefore be based on established efficacy and safety evidence, approved indications, comorbidities, contraindications, reproductive considerations, access, and patient preferences; phenotype-guided matching remains exploratory. Nursing follow-up during pharmacotherapy should assess tolerability, adherence, pregnancy possibility and contraception, and outcomes beyond body weight.

## Long-term maintenance: treating obesity as a chronic, relapsing disease

For most patients, maintaining weight loss is harder than losing weight initially. Compensatory adaptations—including reduced leptin, increased ghrelin, and a sustained increase in subjective appetite—promote weight regain after successful loss. These changes can persist after weight loss, supporting a chronic-care model in appropriate patients [16,23]. Current Korean guidelines recommend maintaining weight loss for at least one year through combined dietary, exercise, and cognitive behavioral therapy, with consideration of continued antiobesity medication supported by long-term safety and efficacy data. Combining lifestyle modification with antiobesity pharmacotherapy is also reasonable for patients who regain weight after bariatric surgery [16].

## Obesity across selected female life stages

The preceding framework of staged assessment, individualized goals, and evidence-based individualized pharmacotherapy applies to all adults with obesity; matching medication to phenotype remains exploratory. In women, treatment must additionally account for the endocrine continuum from the reproductive years through pregnancy and lactation to the menopausal transition. Rather than a single condition, obesity in women is a pathophysiological process continually reshaped by hormonal changes across this continuum.

### Reproductive-age women: polycystic ovary syndrome and insulin resistance

PCOS, a common endocrine disorder in reproductive-age women, frequently coexists with insulin resistance and excess weight. It is not, however, an endocrine cause of obesity and can occur in women without obesity [3]. Higher weight can worsen insulin resistance, hyperandrogenism, and ovulatory dysfunction; lifestyle management and prevention of further weight gain remain central to care. For women with PCOS and higher weight, metabolic and reproductive outcomes can improve with modest weight loss, but the response varies by individual [3]. Metformin may be considered primarily to address metabolic features. For adults with PCOS and higher weight, GLP-1 receptor agonists may be considered alongside active lifestyle intervention in accordance with general population guidelines [3]. Because pregnancy safety data for these agents are lacking, effective contraception is required whenever pregnancy is possible [3]. Given the precautions discussed below, pregnancy status must always be confirmed before any antiobesity medication is initiated in a woman of reproductive age.

### Pregnancy and lactation: safety before weight loss

During pregnancy, the clinical priority shifts from weight loss to appropriate gestational weight gain stratified by BMI. The Institute of Medicine recommends total gains of 12.7–18.1 kg for women who are underweight before pregnancy (BMI <18.5 kg/m^2^), 11.3–15.9 kg for those of normal weight (BMI 18.5–24.9 kg/m^2^), 6.8–11.3 kg for those with overweight (BMI 25.0–29.9 kg/m^2^), and 5.0–9.1 kg for those with obesity (BMI ≥30 kg/m^2^) [5]. These categories use conventional international prepregnancy BMI cutoffs, which differ from the lower threshold for classifying obesity in Korean adults [5,9]. Semaglutide, tirzepatide, and liraglutide should not be used for weight loss during pregnancy. Their current prescribing information states that weight loss provides no benefit and may harm the fetus, and advises discontinuation when pregnancy is recognized [6-8]. Because of its long half-life, semaglutide should be discontinued at least two months before a planned pregnancy [6]. Guidance during lactation remains limited and depends on the agent and formulation. No human milk data are available for subcutaneous semaglutide or liraglutide; oral semaglutide requires separate consideration because its tablet formulation contains an absorption enhancer. The prescribing information for the specific formulation should therefore be consulted [6,8]. In a single-dose study of 11 lactating adults, tirzepatide was undetectable in 164 of 171 milk samples, and the cumulative amount in the remaining samples was less than 0.02% of the maternal dose; its effects on the breastfed infant and milk production remain unknown [7]. These data do not establish infant safety. Decisions during lactation should therefore weigh the developmental benefits of breastfeeding, the mother’s clinical need, and potential effects on the infant, without assuming that one recommendation applies to the entire class. Postpartum weight retention should also be monitored as part of the longer-term weight trajectory [5].

### Tirzepatide and oral contraception: an agent-specific interaction

Clinicians counseling reproductive-age women who receive incretin-based therapy for weight management should emphasize a specific safety issue. Tirzepatide delays gastric emptying, particularly after the first dose, and may reduce the efficacy of oral hormonal contraceptives. For patients using oral hormonal contraceptives, the prescribing information recommends either switching to a nonoral method or adding a barrier method for four weeks after treatment initiation and four weeks after each dose escalation. Nonoral hormonal contraceptives are not expected to be affected [7]. This precaution applies specifically to tirzepatide, rather than to the entire GLP-1 receptor agonist class. Current semaglutide and liraglutide prescribing information has no corresponding instruction for backup contraception, and pharmacokinetic studies found no clinically relevant reduction in overall oral contraceptive exposure [6,8]. Nurses counseling patients at treatment initiation and dose escalation are well positioned to explain the tirzepatide-specific precaution and discuss appropriate nonoral or barrier alternatives.

### The menopausal transition: estrogen withdrawal and visceral fat redistribution

During the menopausal transition, ovarian and chronological aging interact to alter body composition. As circulating estradiol levels decline, fat shifts from subcutaneous to visceral, intra-abdominal depots; this redistribution begins during the transition rather than only after the final menstrual period [4]. Experimental animal studies have suggested that follicle-stimulating hormone (FSH) might affect adiposity independently of estradiol, but their findings are inconsistent. A clinically meaningful effect of rising FSH on adiposity in women therefore remains unestablished [24,25]. Central adiposity can increase during midlife even with only modest changes in total body weight. Both chronological and ovarian aging contribute to changes in body composition, with the menopausal transition particularly associated with central fat redistribution [4,26].

Even without overt weight gain, lean mass can decline as visceral fat increases—a combination associated with greater cardiometabolic risk during midlife [4,26]. Clinical evaluation of postmenopausal women should therefore prioritize waist circumference (abdominal obesity is defined as ≥85 cm in Korean women), assessment of fat and lean mass by BIA or dual-energy X-ray absorptiometry, and metabolic screening with fasting glucose, blood pressure, and a lipid profile. These assessments can detect risks that weight alone would miss [9]. Serial waist circumference measurements and functional assessments may also provide clinically relevant information beyond weight in midlife women.

### Obesity management after menopause: distinct roles of menopausal hormone therapy and glucagon-like peptide-1-based pharmacotherapy

Lifestyle intervention remains the first-line approach, although some patients may not achieve sufficient weight reduction [9,16]. Menopausal hormone therapy (MHT) may modestly limit central fat accumulation, but it should be prescribed for established menopausal indications rather than for weight loss [27]. Before initiating systemic MHT, clinicians should assess for unexplained vaginal bleeding, liver disease, prior estrogen-sensitive cancer, coronary heart disease, stroke, myocardial infarction, venous thromboembolism, and a personal or inherited high risk of thromboembolism [27]. Family history alone warrants individualized risk assessment; it should not be equated with a personal history or a confirmed inherited high-risk state [27]. GLP-1 receptor agonists or dual GLP-1/GIP receptor agonists may be considered for postmenopausal women under their respective obesity indications, including women already receiving MHT. In a small retrospective cohort, MHT users had greater semaglutide-associated weight loss than nonusers; the nonrandomized design, however, does not establish that MHT augments this weight loss [28]. Concurrent treatment should therefore be based on an independent indication for each therapy and individualized assessment, rather than used routinely as a combined strategy.

### Case, part 2

One plausible management course for the illustrative patient introduced earlier is outlined here. Assessment organized with the 4Ms and severity grading with EOSS would identify postmenopausal central obesity with predominantly visceral adiposity, declining lean mass with an increased risk of sarcopenia, and multiple cardiometabolic risk factors: treated dyslipidemia, impaired fasting glucose, and abdominal obesity. Her established dyslipidemia and emerging metabolic abnormalities in the presence of abdominal adiposity would be consistent with EOSS stage 2, which denotes established obesity-related chronic disease or moderate functional limitation. Her dyslipidemia is an established, pharmacologically treated obesity-related chronic disease, placing her beyond stage 1, which denotes only subclinical risk factors or mild symptoms. Neither significant end-organ damage nor functional impairment was documented, so she does not meet the criteria for stage 3 [14]. Staging thus complements the earlier diagnosis of clinical rather than preclinical obesity: the Lancet criteria establish the presence of obesity-related illness, whereas EOSS grades its overall medical, mental, and functional burden [13,14]. Metabolic syndrome could not be formally confirmed because blood pressure and high-density lipoprotein cholesterol were unavailable. Sarcopenia and a phenotype characterized by reduced resting energy expenditure could not be assessed because muscle strength, physical performance, and resting energy expenditure were not measured. With a BMI exceeding 27 kg/m^2^ and coexisting dyslipidemia and impaired fasting glucose, she met the approved indication for antiobesity pharmacotherapy. A reasonable plan could combine semaglutide, resistance exercise at least three times weekly, adequate protein intake, and serial monitoring of waist circumference and lean mass. In this hypothetical illustration, her weight would decrease from 72 kg to 65 kg (a 9.7% reduction) and her waist circumference from 88 cm to 81 cm over six months. Her lipid profile would improve without a meaningful decline in estimated lean mass, consistent with preferential fat loss. This constructed example illustrates how indicated pharmacotherapy, resistance exercise, nutritional support, and monitoring beyond body weight can be combined; it reports no observed outcomes and does not establish that phenotype-matched treatment is superior.

## Life-stage-specific nursing assessment and shared decision-making

Clinical frameworks and pharmacologic advances will not improve outcomes when care is delivered in a stigmatizing environment. Weight stigma in healthcare settings, including subtle, unconscious clinician bias, can discourage patients from seeking care and harm their physical and psychological outcomes [11,12]. At each encounter, nurses can seek permission to discuss weight, use person-first, nonjudgmental language, and use motivational interviewing to identify the patient’s priorities rather than impose a weight-loss agenda [15,29].

A practical nursing assessment should pair the 4Ms with prompts specific to life stage instead of relying on body weight alone. At every stage, nurses can document weight trajectory, BMI and waist circumference, blood pressure and available metabolic results, mobility and function, sleep, mood and eating concerns, medication exposures, and social barriers [9,15]. Before antiobesity medication is started or continued in reproductive-age women, assessment should cover menstrual irregularity or known PCOS, pregnancy possibility and intention, contraception, and planned conception [3,6-8]. During pregnancy, lactation, and the postpartum period, monitoring should focus on gestational weight gain, postpartum weight retention, breastfeeding goals, and medication safety rather than active pharmacologic weight loss [5-8]. During and after the menopausal transition, nurses should monitor waist circumference, cardiometabolic risk, mobility and function, and potential lean mass loss, while keeping MHT counseling distinct from obesity treatment [9,27].

During pharmacotherapy, nurses can confirm that pregnancy and contraception counseling has occurred, reinforce the tirzepatide-specific backup-contraception interval at treatment initiation and dose escalation, explain expected gastrointestinal adverse effects in advance, and report persistent intolerance or other safety concerns to the prescribing clinician [6-8]. Follow-up should record outcomes beyond weight in kilograms, including waist circumference, metabolic measures, function, treatment tolerability, and the patient’s reproductive or menopausal goals. These activities support shared decision-making: clinical evidence and patient-centered communication inform a plan that reflects the patient’s values, preferences, and life circumstances [30]. Nurses’ roles encompass assessment, education, monitoring, and coordination within local scope-of-practice requirements; they do not include independent diagnosis or prescribing.

## Conclusion

Obesity in women should be assessed as a chronic disease, with evaluation extending beyond BMI alone. Confirmation of excess adiposity, diagnosis of obesity-related illness, multidomain assessment with the 4Ms, and severity staging with EOSS offer complementary information for setting individualized, clinically meaningful treatment goals. Phenotype-guided prescribing and an independent effect of rising FSH, however, remain investigational. Reproductive plans, pregnancy and lactation, and the menopausal transition substantially change treatment priorities and medication counseling; these considerations include the tirzepatide-specific contraception precaution and the use of MHT for established menopausal indications rather than weight loss. Across these stages, nurses play a central role in nonstigmatizing assessment, education, safety monitoring, and shared decision-making within their scope of practice.

## Figures and Tables

**Figure 1.**
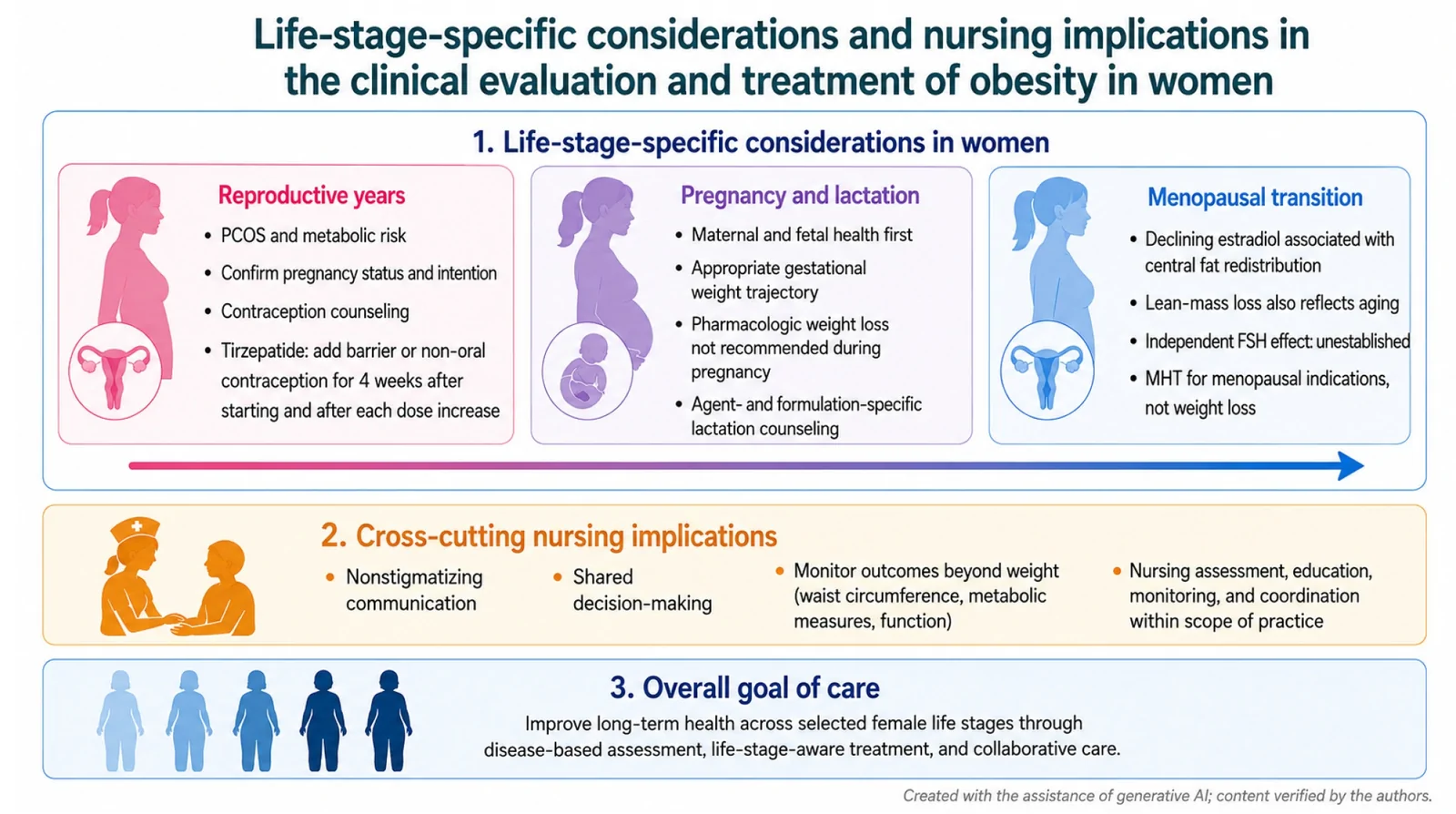
Life-stage-specific considerations and nursing implications in the clinical evaluation and treatment of obesity in women. The figure summarizes considerations for the reproductive years [3,7], pregnancy and lactation [5-8], and the menopausal transition [4,26,27], along with principles applicable across life stages and the overall goal of care [9,11,12,16,30]. The main text describes the general diagnostic and assessment frameworks—the Lancet criteria for clinical obesity [13], the four-domain clinical assessment of mechanical, metabolic, mental, and social milieu factors [15], and the Edmonton Obesity Staging System [14]—which are no longer reproduced in the figure. The authors prepared the figure with a generative artificial intelligence (AI) image tool to assist with illustration and layout; they determined and verified all scientific content and accept full responsibility for its accuracy. FSH: Follicle-stimulating hormone, MHT: menopausal hormone therapy, PCOS: polycystic ovary syndrome.

## References

[1] González-Muniesa P, Mártinez-González MA, Hu FB, Després JP, Matsuzawa Y, Loos RJ (2017). Obesity. Nat Rev Dis Primers.

[2] Cho S, Jung JH, Nam GE, Cho IY, Park KY, Jeong SM (2025). 2024 Obesity fact sheet in Korea: prevalence of obesity, abdominal obesity, obesity defined by body fat percentage, and underweight in adults in Korea from 2013 to 2022. J Obes Metab Syndr.

[3] Teede HJ, Tay CT, Laven JJ, Dokras A, Moran LJ, Piltonen TT (2023). Recommendations from the 2023 international evidence-based guideline for the assessment and management of polycystic ovary syndrome. J Clin Endocrinol Metab.

[4] Sowers M, Zheng H, Tomey K, Karvonen-Gutierrez C, Jannausch M, Li X (2007). Changes in body composition in women over six years at midlife: ovarian and chronological aging. J Clin Endocrinol Metab.

[6] Novo Nordisk Inc (2026). Wegovy (semaglutide) injection, for subcutaneous use; Wegovy (semaglutide) tablets, for oral use: prescribing information.

[7] Lilly USA, LLC (2026). Zepbound (tirzepatide) injection, for subcutaneous use: prescribing information.

[8] Novo Nordisk Inc (2026). Saxenda (liraglutide) injection, for subcutaneous use: prescribing information.

[9] Bae JH, Haam JH, Jeon E, Kang SY, Song S, Park CY (2025). 2024 Clinical practice guidelines for the diagnosis and pharmacologic treatment of overweight and obesity by the Korean Society for the Study of Obesity. J Obes Metab Syndr.

[10] (2000). Obesity: preventing and managing the global epidemic. Report of a WHO consultation. World Health Organ Tech Rep Ser.

[11] Phelan SM, Burgess DJ, Yeazel MW, Hellerstedt WL, Griffin JM, van Ryn M (2015). Impact of weight bias and stigma on quality of care and outcomes for patients with obesity. Obes Rev.

[12] Rubin R (2019). Addressing medicine’s bias against patients who are overweight. JAMA.

[13] Rubino F, Cummings DE, Eckel RH, Cohen RV, Wilding JP, Brown WA (2025). Definition and diagnostic criteria of clinical obesity. Lancet Diabetes Endocrinol.

[14] Sharma AM, Kushner RF (2009). A proposed clinical staging system for obesity. Int J Obes (Lond).

[15] Wharton S, Lau DC, Vallis M, Sharma AM, Biertho L, Campbell-Scherer D (2020). Obesity in adults: a clinical practice guideline. CMAJ.

[16] Kim KK, Haam JH, Kim BT, Kim EM, Park JH, Rhee SY (2023). Evaluation and treatment of obesity and its comorbidities: 2022 update of clinical practice guidelines for obesity by the Korean Society for the Study of Obesity. J Obes Metab Syndr.

[17] Knowler WC, Barrett-Connor E, Fowler SE, Hamman RF, Lachin JM, Walker EA (2002). Reduction in the incidence of type 2 diabetes with lifestyle intervention or metformin. N Engl J Med.

[18] Wing RR, Lang W, Wadden TA, Safford M, Knowler WC, Bertoni AG (2011). Benefits of modest weight loss in improving cardiovascular risk factors in overweight and obese individuals with type 2 diabetes. Diabetes Care.

[19] Acosta A, Camilleri M, Abu Dayyeh B, Calderon G, Gonzalez D, McRae A (2021). Selection of antiobesity medications based on phenotypes enhances weight loss: a pragmatic trial in an obesity clinic. Obesity (Silver Spring).

[20] (2021). Selection of antiobesity medications based on phenotypes enhances weight loss: a pragmatic trial in an obesity clinic. Obesity (Silver Spring).

[21] Wilding JP, Batterham RL, Calanna S, Davies M, Van Gaal LF, Lingvay I (2021). Once-weekly semaglutide in adults with overweight or obesity. N Engl J Med.

[22] Jastreboff AM, Aronne LJ, Ahmad NN, Wharton S, Connery L, Alves B (2022). Tirzepatide once weekly for the treatment of obesity. N Engl J Med.

[23] Sumithran P, Prendergast LA, Delbridge E, Purcell K, Shulkes A, Kriketos A (2011). Long-term persistence of hormonal adaptations to weight loss. N Engl J Med.

[24] Liu P, Ji Y, Yuen T, Rendina-Ruedy E, DeMambro VE, Dhawan S (2017). Blocking FSH induces thermogenic adipose tissue and reduces body fat. Nature.

[25] Libby AE, Solt CM, Jackman MR, Sherk VD, Foright RM, Johnson GC (2024). Effects of follicle-stimulating hormone on energy balance and tissue metabolic health after loss of ovarian function. Am J Physiol Endocrinol Metab.

[26] Davis SR, Lambrinoudaki I, Lumsden M, Mishra GD, Pal L, Rees M (2015). Menopause. Nat Rev Dis Primers.

[27] “The 2022 Hormone Therapy Position Statement of The North American Menopause Society” Advisory Panel (2022). The 2022 hormone therapy position statement of The North American Menopause Society. Menopause.

[28] Hurtado MD, Tama E, Fansa S, Ghusn W, Anazco D, Acosta A (2024). Weight loss response to semaglutide in postmenopausal women with and without hormone therapy use. Menopause.

[29] Miller WR, Rollnick S (2013). Motivational interviewing: helping people change.

[30] Elwyn G, Frosch D, Thomson R, Joseph-Williams N, Lloyd A, Kinnersley P (2012). Shared decision making: a model for clinical practice. J Gen Intern Med.

